# Pregnancy and Adverse Obstetric Outcomes After Hysteroscopic Resection: A Systematic Review and Meta-Analysis

**DOI:** 10.3389/fsurg.2022.889696

**Published:** 2022-06-27

**Authors:** Xue Wu, Mei Zhang, Ping Sun, Jing-jing Jiang, Lei Yan

**Affiliations:** ^1^ School of Medicine, Cheeloo College of Medicine, Shandong University, Jinan, China; ^2^ Center for Reproductive Medicine, Cheeloo College of Medicine, Shandong University, Jinan, China; ^3^ Qufu Maternity and Infant health Hospital, Qufu, China; ^4^ National Research Center for Assisted Reproductive Technology and Reproductive Genetics, Shandong University, Jinan, China; ^5^ Key laboratory of Reproductive Endocrinology, Ministry of Education, Shandong University, Jinan, China; ^6^ Shandong Provincial Clinical Medicine Research Center for Reproductive Health, Shandong University, Jinan, China

**Keywords:** septate uterus, hysteroscopic resection, septum resection, pregnancy outcomes, live birth rate, term delivery, adverse obstetric outcomes

## Abstract

**Objective:**

Although the randomized controlled trial (RCT) of the efficacy of hysteroscopic resection in women with uterine septum has not shown any significant correlation in recent research, motivation for deeper study remains insufficient. In this study, the objective was to determine pregnancy-related outcomes, along with adverse obstetric outcomes, following hysteroscopic resection and also to determine whether women with hysteroscopic resection bear the same outcomes as women with normal uterine cavities.

**Search Methods:**

From January 1995 to February 2022, a systematic literature review was conducted to identify all studies published concerning the gestation outcomes of women with and without hysteroscopic resection while comparing the gestation outcomes of women after hysteroscopic resection and with a normal uterine cavity. Our primary outcome was the live birth rate (LBR). The secondary outcomes were term delivery, preterm delivery, spontaneous miscarriage, malpresentation, cesarean section, and other adverse obstetric outcomes.

**Results:**

22 studies were included in this meta-analysis. The control groups of 14 studies were treated women, and the control groups of the other 8 studies were patients bearing a normal uterine cavity. Hysteroscopic resection was related to a higher rate of term delivery (OR = 2.26, 95% CI, 1.26–4.05), and a lower rate of spontaneous abortion (OR = 0.50, 95% CI, 0.27–0.93), and a lower rate of malpresentation (OR = 0.31, 95% CI, 0.19–0.50). Nevertheless, in comparison with the normal uterus group, the rates of preterm birth, cesarean section, and postpartum hemorrhage after resection did not return to normal levels.

**Conclusion:**

Hysteroscopic resection can effectively reduce the risk of abortion and malpresentation in patients possessing a uterine septum while increasing the term delivery rate. Although well-designed RCTs should confirm our meta-analysis, it still bears recommending to patients

## Introduction

A septate uterus is regarded as the most frequent type of uterus abnormality, caused by varying degrees of dysfunction of the bilateral adrenal ducts. Accordingly, the uterus fuses between 6–18 weeks of embryonic development. A congenital anomaly, this condition is known to be harmful to fertility. The frequency of septate uterus among fertile women proves to be about 0.2%–2.3% (1). For an extended period, a septate uterus has been associated with recurrent spontaneous abortion (RSA), late abortion, and preterm birth (2–5), as well as cesarean section and fetal malpresentation (6).

Hysteroscopic metroplasty is the standard method for restoring the anatomical structure of the normal uterine cavity (7). As a result, women with septum resection ought to bear similar pregnancy outcomes to women with normal pregnancies (8). According to some studies, fertility will prove highly diminished if malpresentation is not treated. Surgery has improved reproductive outcomes in patients with RSA (9, 10). In a recent scientific impact paper, surgical treatment can be recommended only for women with recurrent miscarriages and uterine septum, which can improve their chances of successful pregnancy (11).

The exact pathophysiological mechanism of the uterine septum associated with infertility remains unclear (12). Studies have shown that hysteroscopic resection cannot cure patients with unexplained infertility (13). An RCT on the efficacy of hysteroscopic metroplasty has just been published, and this RCT and previously substantive collection of cohort studies failed to discover any significant differences in terms of reproductive outcomes (14, 15). But it did not specifically evaluate women scheduled to undergo in vitro fertilization (IVF). As a matter of some concern, the presence of a septa uterus frequently leads to recurrent abortion, although a proportion of patients enjoy normal pregnancy and delivery without symptoms. Some researchers believe that the uterine septum may be a potential risk factor for infertility (10). Studies on reproductive outcomes of women with primary infertility after IVF demonstrate that the abortion rate can be significantly reduced when the operation was performed before IVF (16). In the artificial frozen-thawed embryo transfer (FET) cycles, the clinical pregnancy rate and live birth rate in the operation group proved to be much higher (17). Although many studies have involved patients with unexplained infertility and uterine septum, the impact of the uterine septum on infertility and the indication of surgery remains controversial (18). In addition, the surgical process shows a tendency toward injury, which may incur surgical complications. It was reported that the incidence of uterine perforation after septum surgery was 1.1% (19), and the rate of intrauterine adhesion was 5% (20).

The purpose of any surgical intervention is to restore the anatomical structure of endometrial cavity and cervical canal as much as possible, and to restore the normal volume and shape. After that, normal menstrual flow and sufficient sperm transport are allowed for fertilization and implantation. The American Society of Reproductive Medicine (ASRM) 2016 guidelines make a recommendation for septum resection for patients without infertility or prior pregnancy loss after an evaluation of the potential risks and benefits of surgery (21). Nevertheless, the European Society of Human Reproduction and Embryology (ESHRE) and the Royal College of Obstetricians and Gynaecologists (RCOG) hold that insufficient evidence supports resection and that further research is required to assess this methodadequately (22). A study concluded that removing either endocervical or decidual polyps seems to be associated with an increased risk of pregnancy loss and preterm birth. It is necessary to evaluate the removal of mediastinum before pregnancy because the uterine mediastinum cannot be removed during pregnancy (23). Admittedly, the majority of these studies were retrospective and compared reproductive outcomes before and after hysteroscopic, which inevitably resulted in some bias (14–17). Although the only RCT (15) provided the highest quality evidence, it still bore limitations, such as research design defects and insufficient motivation. Ultimately, a host of clinical questions remain unanswered, and debates are ongoing concerning the management of the condition and whether the patient should be treated.

In our study, we employed a method for better exploring the clinical significance of uterine septum incision. In this regard, we comprehensively compared the two methods: hysteroscopic resection versus no surgery treatment, comparing hysteroscopic resection to the normal uterus. Our study aimed to supply answers to related clinical questions: Is hysteroscopic resection of the septum uterus clinically beneficial for reproductive outcomes? Are variances observed in women with different histories between RSA and primary infertility after hysteroscopic surgery? Are variances observed in terms of obstetric outcomes between women who have undergone surgery and women with a normal uterus? Does the evidence suggest any indication that hysteroscopic hysteroplasty leads to complications during pregnancy or delivery?

## Methods

### Search Methods of Studies

**Figure 1:**
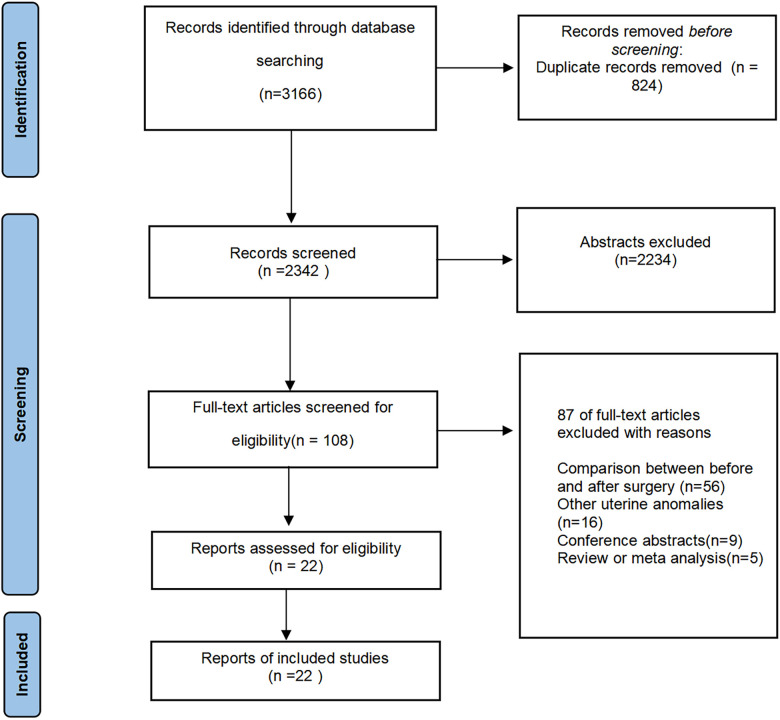
Flow chart.

All published studies on surgical treatment of septum uterus and reproductive outcomes were searched. We also consulted a search Methodist. PUBMED, EMBASE, Cochrane Central Register of Controlled Trials, and Web of Science were electronically searched from January 1995 to February 2022 for studies that compared reproductive outcomes of women with and without hysteroscopic resection, and compared the reproductive outcomes in women after hysteroscopic resection and with a normal uterine. A search strategy was carried out according to the following search string and keywords: “septate uterus”, “hysteroscopic resection”, “septum resection”, “pregnancy outcomes”, “live birth rate”, “term delivery”, “adverse obstetric outcomes”. Case-control studies, and RCTs met the inclusion conditions. Manually searched the list of other resource references for major articles and review articles, and we also manually retrieved relevant conference abstracts. All relevant reports were consulted to identify further articles that could be included in this meta-analysis. Duplicates were automatically or manually recognized and discarded (Figure 1).

### Criteria for Considering Studies

Studies were included if: (i) Cohort (prospective and retrospective), RCTs and case-controlled studies compared the gestation outcomes of patients who have accepted hysteroscopic metroplasty with untreated women and provided results of interest to us; (ii) RCTs, cohort and case-controlled studies compared reproductive outcomes between women who have undergone hysteroscopic hysterectomy and women with the intact uterine cavity.

Studies were excluded if: (i) only operation group without a control group; (ii) comparison of reproductive outcomes before and after operation in the same group of women; (iii) other uterine anomalies, such as the unicornous uterus and arcuate uterus; (iv) case reports or with insufficient information about the results of interest.

### Type of Outcomes

The main outcome was LBR. The secondary outcomes were as follows: (i) Spontaneous miscarriage: spontaneous death before 24 weeks of gestation; (ii) Preterm delivery: born before 37 full weeks of gestation; (iii) Term delivery; (iv) Adverse obstetric outcomes including caesarean section and obstetric complications such as placenta praevia, postpartum hemorrhage, uterine rupture, and placental abruption.

### Selection of Studies

We conducted a preliminary screening of overall titles and abstracts searched by XW, and we also selected all potential full texts that meet the meta-analysis. The full texts of these latent qualified articles were researched and independently assessed by reviewers (XW and PS). Any discrepancies were resolved through discussions among the group review members. The searched data included study characteristics such as demographic data, diagnostic methods, duration of follow-up, and various outcome data.

### Assessment of Heterogeneity

The purpose of the heterogeneity test was to examine whether the results of individual studies could be combined. We used I^2^ to evaluate the heterogeneity of this study. The I^2^ score below 50% was regarded as having low or moderate heterogeneity, while the score of I^2^ equal to or greater than 50% was thought to be highly heterogeneity. We reported risk ratios (RRs) using a random-effects model when there was remarkable heterogeneity in the article. It needed to assume that the estimated effects in different studies were different, but follow a certain distribution.

### Measures of Treatment Effects

Statistical analyses were performed using Review Manager (version 5.4, The Cochrane Collaboration) and STATA (version 9.0, Stata Corp). We used Odds Ratio (OR) with a 95% confidence interval (CI), as pooled effect measures for primary and secondary outcomes. In all analyses, *p* < 0.05 was considered statistically significant. For dichotomy data, the number of events per a study in the experimental and control groups was entered into the Review Manager 5, and OR was used for analyzed analysis, while for continuous data, standardized mean differences (SMD) between the two groups was analyzed.

### Subgroup Analysis

Poor obstetric history might be a major source of heterogeneity, such as RSA and primary infertility. Therefore, we tried our best to measure the combined outcomes of RSA, primary infertility and unclassified, respectively, and to explore the effect of hysteroscopic treatment on the pregnancy results of patients with different pregnancy histories.

### Quality, Sensitivity Analysis, Bias Risk and Publication Bias Assessment

We evaluated the methodological quality of the included studies, in which the quality of non-randomized studies was based on the Newcastle-Ottawa scale (24). According to the eight projects are divided into three areas: the selection of research groups, the comparability of groups and the determination of results. The score for each quality item was represented by a star to provide a visual assessment. Studies will be awarded 9 stars if they complete all high-quality projects. The modified Jadad scale (7 points) was used to evaluate the included RCT. The items included randomization, concealment of allocation, double-blind and withdrawals and dropouts. The only RCT study with a score of 5 was defined as high quality.

**Figure 2:**
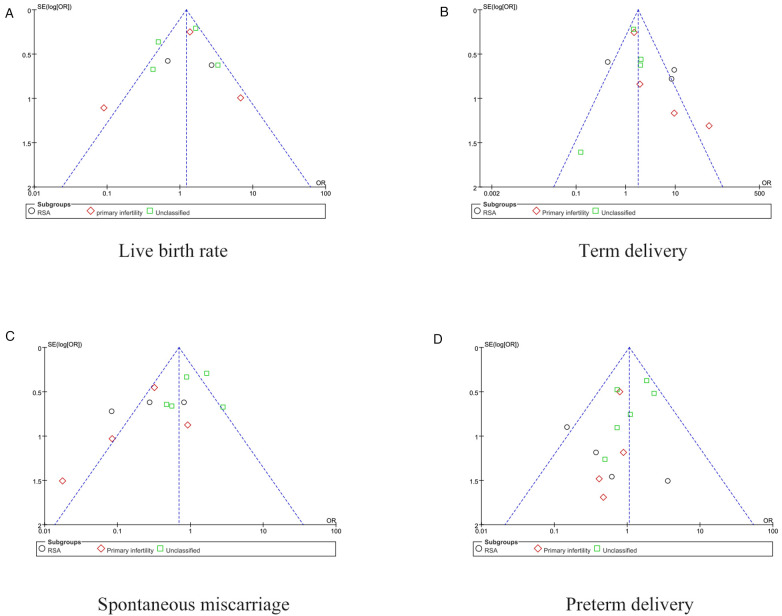
Funnel plot of the meta-analysis for (**A**) Live birth rate, (**B**) Term delivery, (**C**) Spontaneous miscarriage and (**D**) Preterm delivery.

Details of the quality assessment are shown in (Supplementary Table S1). Funnel plots were generated for outcomes involving more than 10 studies to assess publication bias (Figure 2). We conducted a sensitivity analysis on the results of cesarean section with significant heterogeneity. The original qualitative interpretation of the combined results did not change with the transformation between the random effect model and the fixed model. In addition, the overall results of heterogeneity did not decrease obviously when we deleted each of the papers in turn (Table 1).

**Table 1:** Outcomes for sensitivity analysis of included studies evaluating caesarean section.

	OR (95% CI)	Heterogeneity test	
		*p*	I^2^ (%)
(a)			
Chen et al, 2013	1.19 (0.42–3.38)	0.02	69
Fox et al, 2019	0.63 (0.22–1.83)	0.12	49
Heinonen, 1997	1.39 (0.52–3.69)	0.11	50
Rikken et al, 2021	0.72 (0.19–2.80)	0.006	76
Sugiura-Ogasawara et al, 2014	0.87 (0.23–3.28)	0.005	77
(b)			
Agostini, MD* et al, 2009	2.07 (0.78–5.20)	0.02	82
Kenda Šuster et al, 2016	8.09 (1.22–53.65)	0.007	86
Ono et al, 2019	5.08 (0.29–88.61)	<0.0001	94

*(a) The control group of untreated women with septum uterus, and the study group was patients who underwent hysteroscopic surgery. (b) The control group of these articles was women with intact uterus, and the study group was patients who underwent hysteroscopic surgery.*

## Results

The search strategy produced 3,166 studies. 2,234 studies were excluded because their titles and abstracts clearly did not meet the requirements. Of the total 108 potentially relevant manuscripts identifed, 86 studies were excluded after evaluating the full text. The reasons of exclusion include before-and-after controls (*n* = 56) other uterine anomalies (*n* = 16), conference abstracts (*n* = 9), review or meta analys (*n* = 5). Twenty-two studies were included in the meta-analysis.

Most of the included studies were non-RCT. Selected studies include an RCT (15). Twenty-two studies included more than one comparison group, of which 14 were compared to untreated controls and 8 to controls with normal uterine structures. Fourteen of these studies involving 1,690 women, 998 women who had hysteroscopic septum resection and 692 women who did not, were included in the meta-analysis. The remaining eight studies involved 7,814 women, of whom 760 women underwent hysteroscopic surgery, and 7,054 women had normal uteruses. In addition, the analysis was carried out respectively on this basis. Four studies provided data on uterine septum patients with recurrent miscarriage (25–28), and four studies provided data on uterine septum patients with primary infertility (14, 17, 29, 30). The specific characteristics of the included studies are shown in (Supplementary Tables S2).

### Live Birth Rate

**Figure 3:**
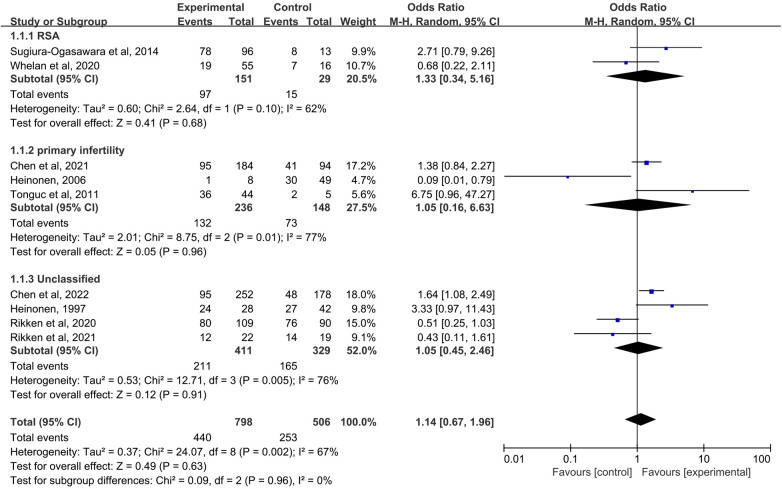
Forest plot of live birth rate for treatment with hysteroscopic metroplasty group versus untreated group.

When the control group was untreated (Figure 3), there was no significant difference in LBR between the resection group and the control group in the RSA subgroup (OR = 1.33, 95% CI, 0.34–5.16; 180 patients; I^2^ = 62%); (25, 27). Similarly, in the primary infertility subgroup, pooled OR for this outcome was also no statistical difference (OR = 1.05, 95% CI, 0.16–6.63; 384 patients; I^2^ = 77%); (16, 29, 31). Four studies analyzed live birth rates and did not distinguish between RSA and primary infertility in comorbidities (14, 15, 17, 29). There was no difference between excised and untreated women (OR = 1.05, 95% CI, 0.45–2.46; 4 studies; I^2^ = 76%); The results were no statistically different when combined (pooled OR = 1.14, 95%-CI, 0.67–1.96; 9 studies, *p* = 0.002, I^2^ = 67%).

### Term Delivery

**Figure 4:**
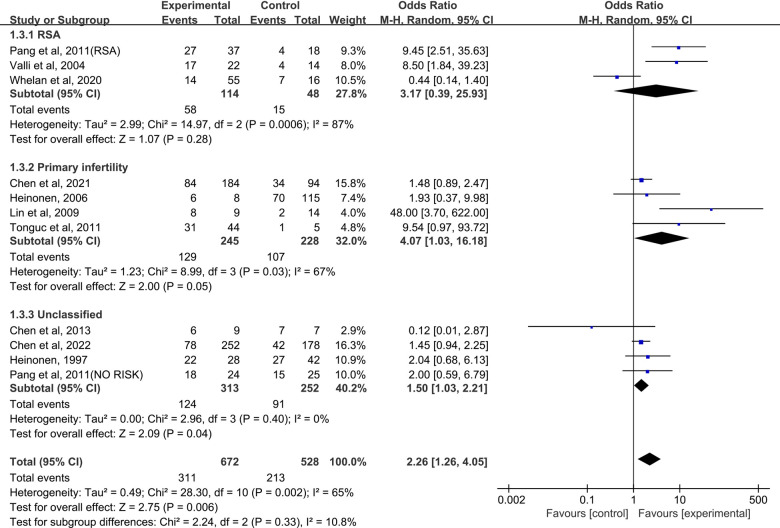
Forest plot of term delivery for treatment with hysteroscopic metroplasty group versus untreated group.

When the control group was untreated (Figure 4), there was no significant difference in term delivery between the resection group and the control group in the RSA subgroup (OR = 3.17, 95% CI, 0.39–25.93; 162 patients; I^2^ = 87%); (25, 26, 28). In the primary infertility subgroup, four studies reported term delivery as an outcome (16, 29–31), and the results showed statistical differences (Pooled OR = 4.07, 95% CI, 1.03 to 16.18; 473 patients; I^2^ = 67%). Four studies analyzed live birth rates and did not distinguish between RSA and primary infertility in comorbidities (17, 26, 32, 33). The significant difference was not found (OR = 1.50, 95% CI, 1.03–2.21; 4 studies; I^2^ = 0%); The results were statistically significant when combined, and the OR = 2.26 (95% CI, 1.26–4.05;11 studies, *p* = 0.002, I^2^ = 65%).

### Preterm Delivery

**Figure 5:**
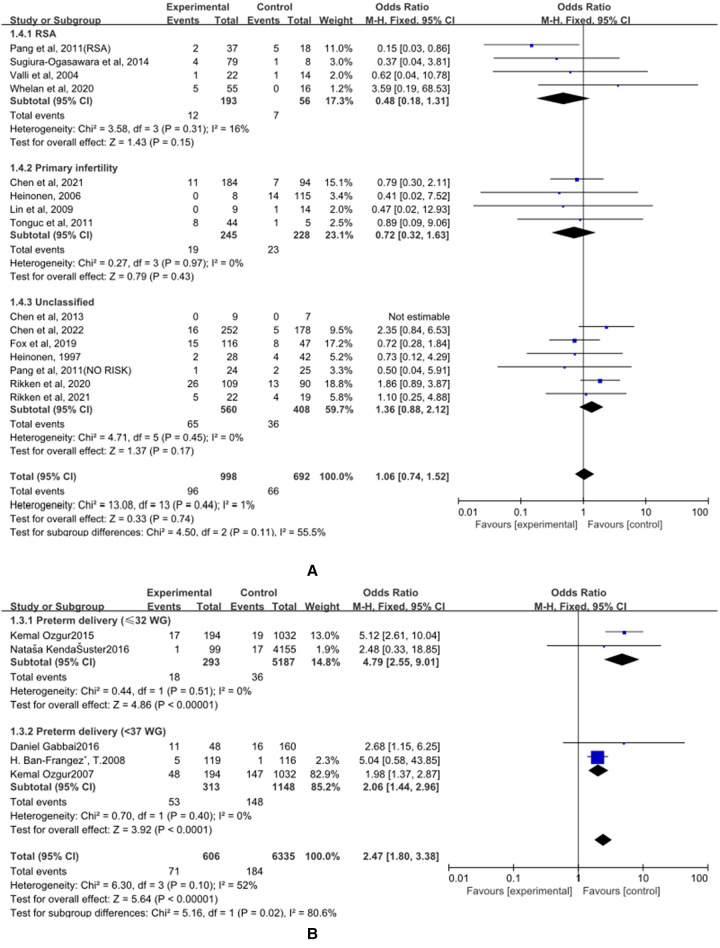
Effect of hysteroscopic resection on preterm delivery. (**A**) The control group of untreated women with septum uterus, and the study group was patients who underwent hysteroscopic surgery. (**B**) The control group of these articles was women with intact uterus, and the study group was patients who underwent hysteroscopic surgery.

(A)The control group of the panel (A) was untreated patients (Figure 5). The study group was patients who had undergone hysteroscopic surgery. Four studies reported this outcome in the RSA subgroup, (25–28) and a significant difference was not found (OR = 0.48, 95% CI, 0.18–1.31; 249 patients; I^2^ = 16%). In the primary infertility subgroup, four studies reported it as an outcome (16, 29–31), and the results showed no statistical differences(OR = 0.72, 95% CI, 0.32–1.63; 473 patients; I^2^ = 0%). Seven studies that did not distinguish between RSA and primary infertility analyzed preterm delivery, and the results did not show significant differences(OR = 1.36, 95% CI, 0.88–2.12; 7 studies; I^2^ = 0%); (14, 15, 17, 26, 32–34). The results were statistically significant when combined, and the OR = 1.06, (95% CI, 0.74–1.52; 14 studies, *p* = 0.45, I^2^ = 0%).

(B) The control group of the panel (B) was women with intact uterine. The study group was patients who had an intact uterus. Two studies have reported this result in the preterm birth (≤32 WG) subgroup, and the results showed statistical differences (OR = 4.79, 95% CI, 2.55–9.01; 5480 patients; I^2^ = 0%); (35, 36). Two studies have reported this result in the preterm birth (<37 WG) subgroup, and the results showed statistical differences (OR = 2.06, 95% CI, 1.44 to 2.96; 1461 patients, *p* = 0.40, I^2^ = 0%); (34, 37). When combined, the results were statistically significant, with OR = 2.47, (95% CI, 1.80–3.38; 4 studies, 6941 patients, *p* = 0.10, I^2^ = 52%).

### Spontaneous Miscarriage

**Figure 6:**
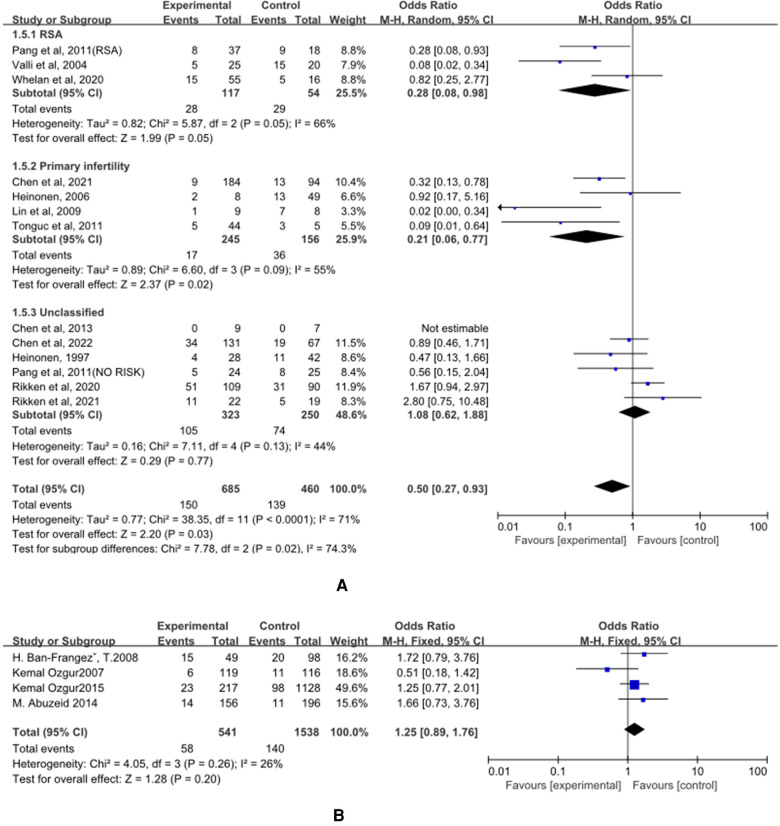
The effect of hysteroscopic metroplasty on spontaneous miscarriage. (**A**) The control group of untreated women with septum uterus, and the study group was patients who underwent hysteroscopic surgery. (**B**) The control group of these articles was women with intact uterus, and the study group was patients who underwent hysteroscopic surgery.

(A) The control group of the panel (A) was untreated patients (Figure 6). The study group was patients who had undergone hysteroscopic surgery. Three studies reported this outcome in the RSA subgroup, (25, 26, 28) and a significant difference was found (OR = 0.28, 95% CI, 0.08–0.98; 3 studies; I^2^ = 56%). In the primary infertility subgroup, four studies reported it as an outcome (16, 29–31) and a significant difference was found. The incidence of women in the study group was significantly lower (OR = 0.21, 95% CI, 0.06–0.77; 401 patients; I^2^ = 55%). Six studies that did not distinguish between RSA and primary infertility analyzed preterm delivery, and the results did not show significant differences (OR = 1.08, 95% CI, 0.62–1.88; 6 studies; I^2^ = 44%); (14, 15, 17, 26, 32, 33) The results were statistically significant when combined, and the OR = 0.50, (95%CI, 0.27–0.93; 13 studies, *p* = 0.03, I^2^ = 71%).

(B) The control group of the panel (B) was women with intact uterine. The study group was patients who have intact uterus. Only four studies looked at spontaneous miscarriage (35, 37–39) and a significant difference was not found OR = 1.25, (95% CI, 0.89–1.76; 2079 patients; *p* = 0.26, I^2^ = 26%)

### Malpresentations

**Figure 7:**
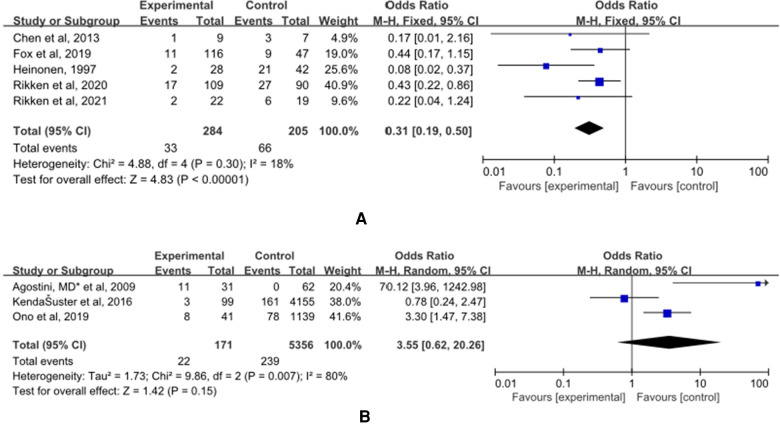
The effect of hysteroscopic metroplasty on malpresentations. (**A**) The control group of untreated women with septum uterus, and the study group was patients who underwent hysteroscopic surgery. (**B**) The control group of these articles was women with intact uterus, and the study group was patients who underwent hysteroscopic surgery.

(A) The control group of the panel (A) was untreated patients (Figure 7). The study group was patients who had undergone hysteroscopic surgery. Five studies reported this outcome (14, 15, 32–34) and a significant difference was found. The incidence of malpresentations in women in the study group was significantly lower (OR = 0.31, 95% CI, 0.19–0.50; 5 studies, 489 patients, *p* = 0.30, I^2^ = 18%).

(B) The control group of the panel (B) was women with intact uterine. The study group was patients who have intact uterus. Five studies reported this outcome (36, 40, 41) and a significant difference was not found OR = 3.55, (95% CI, 0.62–20.26; 5527 patients; *p* = 0.007, I^2^ = 80%).

### Caesarean Section

**Figure 8:**
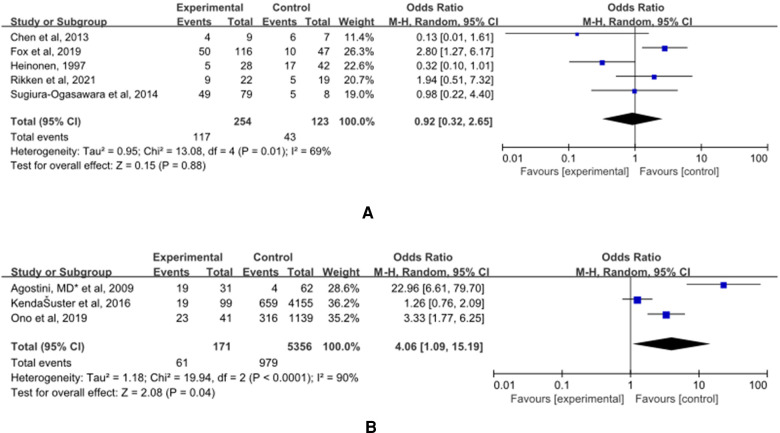
Effect of hysteroscopic metroplasty on caesarean section. (**A**) The control group of untreated women with septum uterus, and the study group was patients who underwent hysteroscopic surgery. (**B**) The control group of these articles was women with intact uterus, and the study group was patients who underwent hysteroscopic surgery.

(A) The control group of the panel (A) was untreated patients (Figure 8). The study group was patients who had undergone hysteroscopic surgery. Five studies reported this outcome (15, 27, 32–34) and a significant difference was not found (OR = 0.92, 95% CI, 0.32–2.65; 377 patients; *p* = 0.01, I^2^ = 69%).

(B) The control group of the panel (B) was women with intact uterine. The study group was patients who have intact uterus. Five studies reported this outcome (36, 40, 41) and a significant difference was found. The incidence of malpresentations in women in the control group was significantly lower (OR = 4.06, 95% CI, 1.09–15.19; 5527 patients; *p* = 0.0001, I^2^ = 90%).

### Other Adverse Obstetric Outcomes

**Figure 9:**
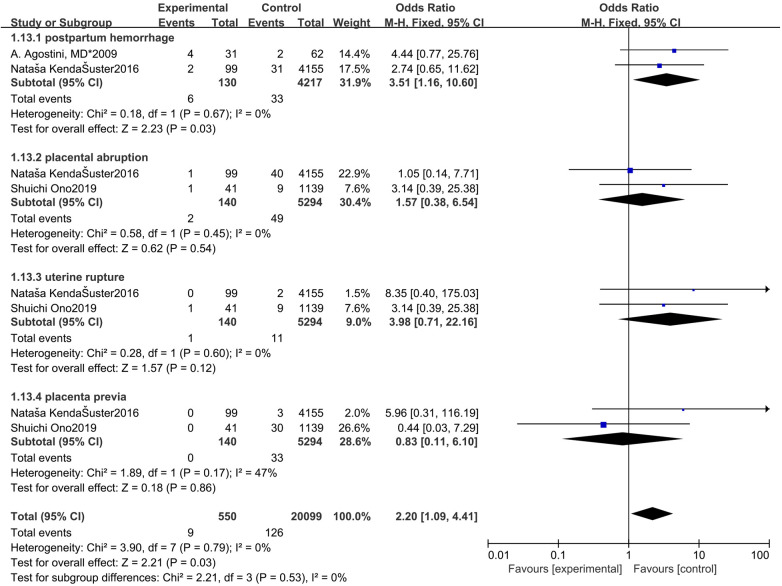
Forest plot of other adverse obstetric outcomes for treatment with hysteroscopic resection group versus intact uterine group.

The control group was women with an intact uterine (Figure 9). The study group was patients who had undergone hysteroscopic surgery. Two studies reported postpartum hemorrhage (36, 41) and a significant difference was found. The incidence of postpartum hemorrhage in women in the control group was significantly lower (OR = 3.51, 95% CI, 1.16–10.60; 4347 patients; *p* = 0.03, I^2^ = 0%). Two studies reported placental abruption (36, 40) and a significant difference was not found. (OR = 1.57, 95% CI, 0.38–6.04; 5434 patients; *p* = 0.45, I^2^ = 0%). Two studies reported uterine rupture (36, 40) and a significant difference was not found (OR = 3.98, 95% CI, 0.71–22.16; 5434 patients; *p* = 0.60, I^2^ = 0%). Two studies reported placenta previa (36, 40) and a significant difference was not found. (OR = 0.83, 95% CI, 0.11–6.10; 5434 patients; *p* = 0.17, I^2^ = 47%).

### Publication Bias

We explored such publication bias using funnel plots (Figure 2). There was no evidence of publication bias in the associations we examined (1, 2 and 3). However, funnel plots 4 showed some asymmetry, which may have led our pooled odds ratios to be overly optimistic. Egger tests were not statistically significant (*p* = 0.288). Therefore, it is unlikely that the observed association was publication bias. Our review has limitations. First, we only examined English peer-reviewed literature, so our study may have missed negative studies in English. Second, studies with a small sample size may have low quality in the implementation process, such as improper randomization, incomplete blindness, and missing follow-up. Finally, accidental factors can also lead to the asymmetry of the funnel.

### Research Conclusions and Heterogeneity

When the control group was untreated women with a uterine septum, the full-term birth rate of patients who underwent hysteroscopy increased, and the rate of abortion and malpresentations decreased. There was no significant difference in the remaining outcomes. The heterogeneity of other studies was high except for preterm delivery and malpresentations. Compared with the intact uterine cavity group, the operation group had higher rates of premature delivery, cesarean section, and postpartum hemorrhage. Except for cesarean section and malpresentations, the heterogeneity of other studies was low.

## Discussion

Our systematic review and meta-analysis demonstrated that hysteroscopic resection is capable of effectively reducing the risk of spontaneous abortion and malpresentation in women with a uterine septum and of returning them to the average population. At the same time, it increased the possibility of full-term delivery. In fact, this trend was increasingly evident when further analyzing the research data on RSA and primary infertility. Although the rates of preterm delivery, cesarean section, and postpartum hemorrhage after hysteroscopy did not return to normal levels, significant evidence indicates that hysteroscopic resection can benefit women with RSA or primary infertility.

Sparse vascular distribution of septum tissue, disorganized and dense smooth muscle structure, the low expression level of estrogen and progesterone receptors, proved to be abnormal ultrastructure in septum covering endometrium were the main reasons affecting pregnancy outcomes (12). In a recent study, previous hysteroscopic resection in primary infertile women could not be considered as a risk factor for preterm birth in singleton pregnancy regardless of the mode of conception, which is similar to our results (42). Our study believe that there is no significant difference in the preterm birth rate between RSA and primary infertility women. Compared with patients with normal anatomy, the rate of spontaneous abortion and fetus in patients undergoing surgery have recovered, but the rate of premature birth and cesarean section is still high. It is not excluded that there are unknown factors, such as the influence of genetic factors. Considering that cervical dilatation before the introduction of surgical hysteroscopy may damage cervical fibers and lead to a high early birth rate (43). In order to minimize the risk of cervical damage, to date it is possible to use thinner instruments (so called miniresectoscopes) that can be used without prior cervical dilatation (44).

Preterm birth itself increases the probability of cesarean delivery, and a history of adverse pregnancy and childbirth outcomes may also influence this probability (18). In addition to the concern over the potentially increased risk of uterine rupture, it remained unclear how surgery increases the risk of cesarean section. Noteworthy, myometrial damage is believed to be the most relevant predisposing factor for uterine rupture, particularly in case of electrosurgery. However, the incidence of uterine rupture during pregnancy is very low (45). This would support the operation safety of subsequent pregnancy. So selective caesarean section should not be recommended.

Intrauterine adhesions is the main long-term complication after caesarean section. Hysteroscopy is also currently considered the gold standard diagnostic and therapeutic approach of patients with intrauterine adhesions. Following hysteroscopic adhesiolysis, intrauterine devices (IUD), stents, or balloon catheters are frequently used to reduce the rate of postoperative adhesion formation, although there is limited data regarding the effect on preventing recurrence of Intrauterine adhesions and subsequent fertility outcomes when these barriers are used (46).

A statistical difference in postpartum hemorrhage rates between women who underwent hysteroscopic surgery and women with normal uterine cavities should be noted. The causes of these differences may be attributable to several aspects. Firstly, hysteroscopic removal of the septum uterus may compromise the integrity of the myometrium in some way and harm contractile strength (47). Secondly, women who undergo cesarean delivery proved at risk of postpartum hemorrhage (48) Thirdly, an intact uterine septum may be accompanied by a vaginal septum, and vaginal delivery can cause a septum tear, which can increase bleeding. Genital tract lacerations prove to be the second leading cause of postpartum hemorrhaging (47).

A meta-analysis found that the pregnancy rate of women who underwent stereoscopic lipectomy was 63.5%, and the live birth rate was 50.2% (49). Venetis et al. performed a meta-analysis of congenital uterine anomalies (CUA) and concluded that hysteroscopic resection was associated with a reduced rate of spontaneous miscarriage (RR = 0.37, 95% CI, 0.25–0.55) (50). As expected, our review also confirmed that hysterectomy was associated with a significant reduction in the incidence of spontaneous abortion and further demonstrated that spontaneous abortion and malpresentation decreased to a frequency exhibited by the greater population. On top of that, our subgroup analysis further demonstrated that hysteroscopic resection reduced the rate of abortion in women with primary infertility or RSA. Nonetheless, for unclassified women, a significant difference in the abortion rate was not evident. It should be noted that retained products of conception is easy to occur in missed abortion caused by septum uterus, which is recommended for surgical treatment (51).

A meta-analysis recently published by Carrera M demonstrated that hysteroscopic resection could effectively reduce the risk of abortion in patients with a complete or partial uterine septum. Further, patients with complete hysterectomy bore a lower risk of abortion, indicating a dose-response gradient in terms of the impact of hysterectomy on reproductive outcomes (52). In the previous meta-analysis, no significant effect of surgery on preterm birth was unearthed, and full-term birth was not analyzed as a reproductive outcome. One essential finding in our study was that hysteroscopic surgery could improve the full-term birth rate of women with a uterine septum. In addition, subgroup analysis showed that this difference derived from the subgroup of primary infertility, a conclusion similar to that of another study (16). Heinonen’s study suggests that patients with unexplained infertility may be subject to other factors leading to infertility (14). As a consequence, other treatments should be considered. For example, for patients with primary infertility, assisted reproductive technology is a promising option (31). In sum, we demonstrated the value of hysteroscopic resection in women with primary infertility. We recommend removal before IVF or intracytoplasmic sperm injection (ICSI) cycles to improve the reproductive outcome of patients.

During this meta-analysis, we possessed the following advantages and innovations: (1) We conducted two comparisons and comprehensive analyses, patients who underwent hysteroscopic resection versus untreated patients; and patients who underwent hysteroscopic resection versus women with normal population; (2) We performed a subgroup analysis of women with RSA along with women with primary infertility; (3) We included pregnancy complications and neonatal indicators. These additions were not seen in previous meta-analyses.

Nevertheless, our study also encountered a series of limitations. The central shortcoming of these studies was that they were almost entirely retrospective, reflecting the practice of a single center. As such, the role of confounding factors and risk may introduce bias. Although the only RCT provided the highest quality evidence, it bore limitations, including a flawed study design, insufficient motivation, and no specific evaluation of women planning IVF. Another possible limitation was that the study adopted variant uterine anomaly classification systems and different diagnostic methods. The literature we searched was published between 1995 and 2022. Significant progress has been achieved in surgical technology, which will bring bias to the results.

Overall, this systematic review demonstrated the potential benefits of hysteroscopic resection for women with a history of adverse obstetric outcomes. Moreover, subgroup analysis demonstrated a higher association with women possessing a history of primary infertility. In point of fact, the vast majority of current studies treat all septum uteri as a single group, although septal subtypes, based on anatomical, radiological, histological, or cellular features, may be extant, producing potentially various effects on fertility and pregnancy outcomes. In the future, based on specific issues, a personalized approach may be required. Despite the existence of the only RCT, the above discussion suggests that we should refocus the uterine septum on reproductive outcomes and that a better-designed, more robust, prospective trial to assess the value of surgical resection is imperative.

## Conclusions

As compared with the untreated control group, hysteroscopic metroplasty was demonstrated to reduce the probability of a spontaneous miscarriage, and malpresentation, while increasing the probability of term delivery rate, especially in women with primary infertility. Compared with the normal uterus group, women with hysteroplasty evinced a higher rate of preterm delivery, cesarean delivery, and postpartum hemorrhage, while hysteroscopic metroplasty was demonstrated to normalize the spontaneous miscarriage and malpresentation in comparison to the healthy controls; meanwhile, other adverse obstetric outcomes proved similar. Eventually, we concluded that hysteroscopic metroplasty ought to be recommended for women with a septum uterus, especially in women with a prior diagnosis of RSA or primary infertility.

## Data Availability

The original contributions presented in the study are included in the article/Supplementary Material, further inquiries can be directed to the corresponding author/s.
