# Supplementary material for: Pregnancy and Adverse Obstetric Outcomes After Hysteroscopic Resection: A Systematic Review and Meta-Analysis
Source: Front Surg. 2022 Jun 27;9:889696. doi: 10.3389/fsurg.2022.889696 (PMC9271824; doi:10.3389/fsurg.2022.889696)
Supplement: Supplementary file 1 [file Table_1_v1.docx]

Supplementary Material

# Supplementary Tables

Supplementary Table 1. Newcastle–Ottawa scale for quality and risk of bias assessment of included studies

| Study | Selection | | | | | | Comparability | | Outcome | | | | | |
| --- | --- | --- | --- | --- | --- | --- | --- | --- | --- | --- | --- | --- | --- | --- |
|  | Representative hysteroscopic metroplasty group | Representative no-surgery group/normal uterine cavity group | | Ascertainment of treatment | | Outcome present at start of study | Comparability of cohorts on the basis of the design or analysis | | Assessment of outcome | | Adequate follow-up | Complete follow-up | | Quality score |
| (a) | | | | | | | | | | | | | | |
| Chen et al, 2013 | - | - | | ★ | | ★ | ★ | | ★ | | ★ | ★ | | 6 |
| Chen et al, 2021 | ★ | ★ | | ★ | | ★ | ★ | | ★ | | ★ | ★ | | 8 |
| Fox et al, 2019 | - | - | | ★ | | ★ | ★ | | ★ | | ★ | ★ | | 6 |
| Heinonen, 1997 | - | ★ | | ★ | | ★ | ★★ | | ★ | | - | - | | 6 |
| Heinonen, 2006 | - | - | | ★ | | ★ | ★ | | ★ | | ★ | ★ | | 6 |
| Lin et al, 2009 | - | - | | ★ | | ★ | ★ | | ★ | | ★ | - | | 5 |
| Pang et al, 2011 | ★ | ★ | | - | | ★ | ★★ | | ★ | | ★ | ★ | | 8 |
| Rikken et al, 2020 | ★ | ★ | | ★ | | ★ | ★ | | ★ | | ★ | ★ | | 8 |
| Chen et al, 2022 | ★ | ★ | | ★ | | ★ | ★ | | ★ | | ★ | ★ | | 8 |
| Sugiura-Ogasawara et al, 2014 | ★ | ★ | | ★ | | ★ | ★ | | ★ | | ★ | ★ | | 8 |
| Tonguc et al, 2011 | ★ | - | | ★ | | ★ | ★ | | ★ | | ★ | - | | 6 |
| Valli et al, 2004 | ★ | ★ | | - | | ★ | ★★ | | ★ | | ★ | ★ | | 8 |
| Whelan et al, 2020 | ★ | ★ | | ★ | | ★ | ★ | | ★ | | - | ★ | | 7 |
| (b) | | | | | | | | | | | | | | |
| Agostini, MD* et al, 2009 | ★ | ★ | | ★ | | ★ | ★★ | | ★ | | - | - | | 7 |
| Ban-Frangezˇ et al, T.2008 | - | ★ | | ★ | | ★ | ★ | | - | | ★ | - | | 5 |
| Ozgur et al, 2007 | ★ | ★ | | ★ | | ★ | ★★ | | ★ | | - | - | | 7 |
| Ozgur et al, 2015 | ★ | ★ | | ★ | | ★ | ★ | | ★ | | - | - | | 6 |
| Abuzeid et al, 2014 | ★ | ★ | | ★ | | ★ | ★★ | | ★ | | ★ | - | | 8 |
| KendaŠuster et al, 2016 | ★ | ★ | | ★ | | ★ | ★★ | | ★ | | - | - | | 7 |
| Ono et al, 2019 | ★ | ★ | | ★ | | ★ | ★★ | | ★ | | ★ | - | | 8 |
| Tomazˇevicˇ* et al, 2010 | - | ★ | | ★ | | ★ | ★★ | | ★ | | - | - | | 6 |
| References | Jadad score | | | | | | | | | | | | | |
|  | Randomization | | Appropriateness of randomization | | Double blind | | | Appropriateness of double blind | | The analysis reasons for withdrawals | | | Sum of jadad Score | |
| Rikken et al, 2021 | 1 | | 1 | | 0 | | | 0 | | 1 | | | 3 | |

(a)These studies compared pregnancy outcomes in women who underwent hysteroscopic septoplasty with those who had no surgery treatment.

(b)These studies compared pregnancy outcomes in women who underwent hysteroscopic septoplasty with those who had normal uterine cavity.

**Supplementary Figure 2.** Characteristics of included studies in the meta-analysis (a)

| Study | Type of Study | | period | | | total  (n=) | | Study vs  control (n =) | | Study population | | Septate uterus  diagnostic method | | | Diagnostic  criteria | Outcomes | | Follow up | |
| --- | --- | --- | --- | --- | --- | --- | --- | --- | --- | --- | --- | --- | --- | --- | --- | --- | --- | --- | --- |
| Chen2013 | Retrospective | | 1997-2010 | | | 21 | | 11/10 | | Un[classified](javascript:;) | | 2DTVS,hysterotomy, hysterotomy during CS, | | | not described | miscarriage or infertility | | 6-24  months | |
| Chen2021 | Retrospective | | 2011-2019 | | | 278 | | 184/94 | | Primary  infertillity | | hysteroscopy,  HSG, 2DTVS,  3DTVS | | | not described | miscarriage, term birth,  preterm birth | | not described | |
| Fox 2019 | Retrospective | | 2005-2018 | | | 163 | | 116/47 | | Un[classified](javascript:;) | | not described | | | not described | term and preterm  birth/CS | | not described | |
| Heinonen1997 | Retrospective | | 1986-1995 | | | 70 | | 28/42 | | Un[classified](javascript:;) | | not described | | | not described | term and preterm  birth/CS/ malpresentation  /CS/ miscarriage | | over years | |
| Heinonen2006 | Retrospective | | 1962-2000 | | | 123 | | 8/115 | | Primary  infertillity | | Laparotomy,  laparoscope, Hysteroscopy, HSG  2DTVS,MRI | | | Not described | Preterm  Cervical cerclage  Gestational hypertension,  Breech,  /CS/Birth weight | | at least 48months | |
| Lin 2009 | Prospective | | 1998-2007 | | | 14 | | 9/7 | | Primary  infertility | | 3DTVS and  HSG | | | Not described | Term and preterm birth/  miscarriage/ | | 6 months to 9 years | |
| Pang 2011 | Prospective | | 2006-2011 | | | 60  78 | | 46/32  30/30 | | 60: RSA  78:NO RISK | | 3DTVS | | | ASRM | RSA/preterm birth | | 15  months | |
| Rikken 2020 | | Retrospective | | 2000-2018 | 257 | | 151/106 | | Un[classified](javascript:;) | | HSG, 3DTVS,  MRI, SHG,  histeroscopy | | ASRM | term birth/preterm birth./ miscarriage/  malpresentation | | | 46  months | |  |

**Supplementary Figure 2.** Continued (a)

| Study | Type of Study | period | total  (n=) | Study vs  control (n =) | Study population | Septate uterus  diagnostic method | Diagnostic  criteria | Outcomes | Follow up |  |
| --- | --- | --- | --- | --- | --- | --- | --- | --- | --- | --- |
| Rikken 2021 | RCT | 2010-2018 | 80 | 40/40 | Un[classified](javascript:;) | HSG, 3DTVS,  MRI, SHSG.  hysteroscopy with  laparoscopy | AFS, 1988;  Grimbizis  2013;  ASRM | LBR/  OPR/CPR/miscarriag  /Preterm birth. | 12  months |  |
| Sugiura-Ogasawara2014 | Prospective | 2003-2009 | 88 | 79/8 | RSA | HSG,2DTVS  Lap,laparotomy,MRI | American  Fertility Society classification of Müllerian anomalies | Preterm birth  Low birth weight /  CS | not described |  |
| Tonguc 2011 | Retrospective | 2006-2009 | 127 | 102/25. | Primary  infertillity | HSG | ASRM | CPR/miscarriage,  preterm birth/, term birth. | 14  months |  |
| Valli 2014 | Prospective | 1990-2001 | 48 | 23/15 | RSA | hysteroscopy | ASRM | CPR  Term birth/preterm birth /Miscarriage. | 36  months |  |
| Whelan 2020 | Prospective | 2004-2012 | 32 | 21/11 | RSA | 3DTVS,  hysteroscopy | Not described | LBR/Preterm Birth/ miscarriage | not described |  |
| Chen 2022 | Prospective | 2009-2019 | 430 | 252/178 | Secondary  infertillity | 3DTVS,  hysteroscopy | Not described | cumulative pregnancy  Rate/cumulative live birth rate/CPR/LBR/ biochemical pregnancy rate/premature  live birth rate/term delivery rate/post-term delivery rate/ectopic pregnancy rate/ miscarriage rate | not described |  |
|  |  |  |  |  |  |  |  |  |  |  |
|  |  |  |  |  |  |  |  |  |  |  |

**Supplementary Figure 2.** Characteristics of included studies in the meta-analysis (b)

| Study | Type of Study | period | total  (n =) | Study vs  control (n =) | Study population | Surgical method | Outcomes | Follow up |  | |  | |
| --- | --- | --- | --- | --- | --- | --- | --- | --- | --- | --- | --- | --- |
| A.Agostini, MD*2009 | Retrospective | 1996-2004 | 93 | 31/62 | history of hysteroscopic metroplasty VS normal uterus had term pregnancies | A 7-mm operative hysteroscope with  a pointed monopolar electrode | fetal presentation, gestational age at delivery, rate of  caesarean section, and subsequent evacuation of uterus)/ birth weight | not described |  |  | |  |
| H.Ban-Frangezˇ, T.2008 | Retrospective | 1993-2004 | 147 | 49/98 | Large septum after resection VS normal uterus | using a continuous  flow resectoscope | Abortion | not described |  |  | |  |
| Kemal Ozgur2007 | Retrospective | 2002-2004 | 235 | 119/116 | incomplete  uterine septum after resection VS normal uterus | an L type needle coagulation  electrode was used with a hysteroscopic resectoscope | Clinical pregnancies /Pregnancy losses Multiple pregnancies/  Preterm labour | not described |  |  | |  |
| Kemal Ozgur2015 | Retrospective | 2005-2012 | 1345 | 217/1128 | incomplete  uterine septum after resection VS no intrauterine anomalies. | using a 26 French resectoscope and a  monopolar, 90° angled, cutting knife electrode | CPR /miscarriage,  preterm birth/LBR/ Stillbirth rate /birth weight | not described |  |  | |  |

**Supplementary Figure 2.** Continued (b)

| Study | Type of Study | period | total  (n =) | Study vs  control (n =) | Study population | Surgical method | Outcomes | Follow up | |  | |  | |
| --- | --- | --- | --- | --- | --- | --- | --- | --- | --- | --- | --- | --- | --- |
| M.Abuzeid 2014 | Retrospective | 1992-2011 | 352 | 156/196 | following hysteroscopic septoplasty of an incomplete uterine septum or arcuate  anomaly VS normal endometrial cavity | solid hysteroscopic  scissors and operative hysteroscope were used | Clinical pregnancy Delivered/Miscarried/Number Ectopic | not described | |  |  | |  |
| Nataša KendaŠuster  2016 | Retrospective | 2006-2011 | 4254 | 99/4155 | underwent hysteroscopic metroplasty VS normal endometrial | An 8-mm monopolar Karl Storz resectoscope | Preterm labor, hemorrhage before and after delivery, mean weeks of gestation at delivery, mean birth weight, breech and CS | not described | |  |  | |  |
| Shuichi Ono2019 | Retrospective | 2011-2016 | 1180 | 41/1139 | underwent hysteroscopic metroplasty using the incision method for septate uterus VS who delivered with normal endometrial during  the same period | a 26-Fr resectoscope | mean weeks of delivery, mean birthweight/CS/breech /post-partum hemorrhage, preterm delivery/placental  Abruption/placenta previa, /placenta accreta and uterine rupture | not described |  |  |  | |  |
| Tomazˇevicˇ*2010 | Retrospective | 1993-2015 | 825 | 275/550 | Large septum terus after resection VS women with a normal uterus | a continuous flow  resectoscope | Pregnancy/Live birth | not described |  |  |  | |  |

(a)These studies compared pregnancy outcomes in women who underwent hysteroscopic septoplasty with those who had no surgery treatment.

(b)These studies compared pregnancy outcomes in women who underwent hysteroscopic septoplasty with those who had normal uterine cavity.

MRI, magnetic resonance imaging; DTVS, dimensional transvaginal sonography; CS, cesarean section; RSA, recurrent spontaneous abortion; NO RISK, women were randomly divided in this study; ASRM, The American Society of Reproductive Medicine; 2DTVS, Bidimensional transvaginal sonography; 3DTVS, Tridimensional transvaginal sonography; CPR, clinical pregnancy rate; LBR= live birth rate; HSG, hysterosalpingography; OPR, ongoing pregnancy rate; PE, preeclampsia; SHG, sonohysterography.
